# Highly Contiguous Genome Assemblies of 15 *Drosophila* Species Generated Using Nanopore Sequencing

**DOI:** 10.1534/g3.118.200160

**Published:** 2018-08-07

**Authors:** Danny E. Miller, Cynthia Staber, Julia Zeitlinger, R. Scott Hawley

**Affiliations:** * Stowers Institute for Medical Research, Kansas City, MO; † MD-PhD Physician Scientist Training Program, University of Kansas Medical Center, Kansas City, KS; ‡ Department of Pathology, University of Kansas Medical Center, Kansas City, KS 66160; § Department of Molecular and Integrative Physiology, University of Kansas Medical Center, Kansas City, KS

**Keywords:** Genome assembly, Drosophila, Nanopore sequencing, third-generation sequencing

## Abstract

The *Drosophila* genus is a unique group containing a wide range of species that occupy diverse ecosystems. In addition to the most widely studied species, *Drosophila melanogaster*, many other members in this genus also possess a well-developed set of genetic tools. Indeed, high-quality genomes exist for several species within the genus, facilitating studies of the function and evolution of *cis*-regulatory regions and proteins by allowing comparisons across at least 50 million years of evolution. Yet, the available genomes still fail to capture much of the substantial genetic diversity within the *Drosophila* genus. We have therefore tested protocols to rapidly and inexpensively sequence and assemble the genome from any Drosophila species using single-molecule sequencing technology from Oxford Nanopore. Here, we use this technology to present highly contiguous genome assemblies of 15 Drosophila species: 10 of the 12 originally sequenced Drosophila species (*ananassae*, *erecta*, *mojavensis*, *persimilis*, *pseudoobscura*, *sechellia*, *simulans*, *virilis*, *willistoni*, and *yakuba*), four additional species that had previously reported assemblies (*biarmipes*, *bipectinata*, *eugracilis*, and *mauritiana*), and one novel assembly (*triauraria*). Genomes were generated from an average of 29x depth-of-coverage data that after assembly resulted in an average contig N50 of 4.4 Mb. Subsequent alignment of contigs from the published reference genomes demonstrates that our assemblies could be used to close over 60% of the gaps present in the currently published reference genomes. Importantly, the materials and reagents cost for each genome was approximately $1,000 (USD). This study demonstrates the power and cost-effectiveness of long-read sequencing for genome assembly in Drosophila and provides a framework for the affordable sequencing and assembly of additional Drosophila genomes.

The early availability of high-quality genome assemblies for 12 species of Drosophila fostered many studies in evolution and comparative genomics, reinforcing Drosophila’s role as a primary model organism (Adams *et al.* 2000; Drosophila 12 Genomes Consortium *et al.* 2007). Since the publication of these sequences, improvements have been made to the original 12 genomes (Hoskins *et al.* 2015) and the genomes for several additional species have been reported (c. f. Ometto *et al.* 2013; Nolte *et al.* 2013; Chiu *et al.* 2013; Allen *et al.* 2017). Although the quality and value of these genomes is high, the cost and effort required to assemble new genomes remains prohibitive for many laboratories. These issues, as well as the difficulty of assembling repetitive or low-complexity regions using short-read technology alone, must be overcome before we can rapidly increase the number of sequenced species.

Long-read, or third-generation, sequencing technology promises to simplify genome assembly by generating individual reads longer than many of the repetitive or low-complexity regions that have complicated genome assembly in the past (Chaisson *et al.* 2015). Indeed, long-read data for the *Drosophila melanogaster* reference genome stock ISO-1 generated on a Pacific Biosciences RSII were released in 2014 (Kim *et al.* 2014). These data were used to assemble a high-quality *D. melanogaster* genome with a contig N50 of 21 Mb (contig N50 is a measure of genome continuity in which half of the genome is contained in overlapping DNA segments, or contigs, larger than the value given), demonstrating that long reads could be used to generate highly contiguous genome assemblies in Drosophila (Berlin *et al.* 2015).

Genome assemblies using Oxford Nanopore sequencing technology have been reported for several species, including *Saccharomyces cerevisiae* (Salazar *et al.* 2017), *Arabidopsis thaliana* (Michael *et al.* 2017), *Caenorhabditis elegans* (Tyson *et al.* 2017), and *Homo sapiens* (Jain *et al.* 2018). This technology measures changes in current as a molecule (currently either DNA or RNA) passes through a small pore in a membrane. It has several advantages over other long-read technologies, including low DNA input requirements, ease of library preparation, and no theoretical limit to the length of a sequencing read. Although there are concerns about a high error rate with this technology, there are methods to mitigate this concern (Walker *et al.* 2014; Simpson *et al.* 2017; Vaser *et al.* 2017). For example, polishing using the original long-read data or data from another technology such as Illumina can correct SNP and indel errors by aligning reads to the assembled genome and identifying sites where modifications to the assembly result in resolution of a SNP or indel (Walker *et al.* 2014; Simpson *et al.* 2017). The cost of Nanopore sequencing is also attractive for small to medium-sized genome assembly—as of early 2018, up to 15 Gb of data could be generated on a single flow cell under ideal conditions for approximately $1,000 USD. Because of the advantages and the relatively low cost, we wondered if we could create a highly contigious, non-scaffolded genome assembly from reads generated using a single flow cell.

Here, we report the sequencing and assembly of 15 non-melanogaster Drosophila species: ananassae, biarmipes, bipectinata, erecta, eugracilis, mauritiana, mojavensis, persimilis, pseudoobscura, sechellia, simulans, triauraria, virilis, willistoni, and yakuba (Figure 1). Nanopore sequencing and assembly of D. melanogaster, including genome scaffolding using additional technologies, is presented in a co-submitted manuscript (Solares *et al.* 2018). These 15 species were sequenced to an average depth of coverage of 29x and an average read length of 5.9 kb. We rapidly assembled each genome using miniasm (Li 2016; 2018), resulting in an average contig N50 of 4.4 Mb. Assemblies were polished using either Nanopore data alone, Illumina short-read data alone, or a combination of both. Polishing with both Nanopore and Illumina data resulted in genomes containing on average 97.7% of all single-copy genes expected to be present in metazoans, consistent with current Drosophila reference assemblies. In addition, for the 10 species included here as part of our analysis of the original 12 genomes project, 97.8% of transcripts either fully or partially aligned to our assembled contigs, only 1.7% fewer transcripts than aligned to the current published reference genomes.

**Figure 1:**
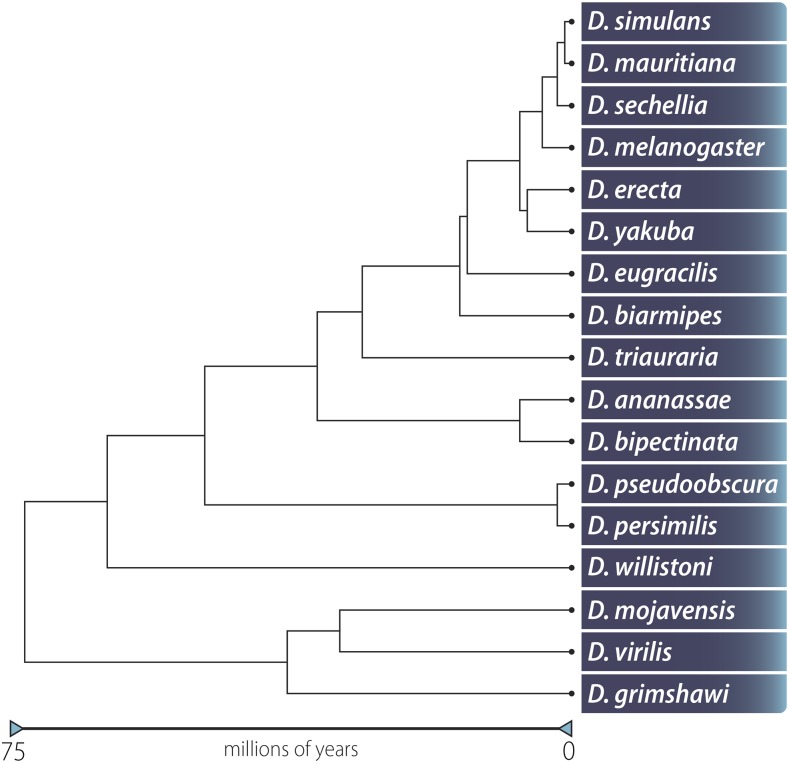
Phylogenetic tree of flies sequenced in this report including two species (*D. melanogaster* and *D. grimshawi*) not sequenced here but that were part of the original 12 genomes project (Drosophila 12 Genomes Consortium *et al.* 2007). Adapted from Thomas and Hahn (2017).

Finally, after mapping contigs from currently published reference genomes to determine how many gaps could be closed using our highly contiguous assemblies, we estimate that an average of 61% of gaps in the currently published reference genomes could be resolved using our data. While each genome was highly contiguous, the overall materials and reagents cost was relatively low at approximately $1,000 USD per genome. This study demonstrates that it is feasible to generate a highly contiguous, low-cost genome for any member of the Drosophila genus and provides a framework by which additional Drosophila genomes may be assembled.

## Materials and Methods

### Stocks and reference genomes

All stocks used in this study (Figure 1, Table 1) are available at the National Drosophila Species Stock Center (http://blogs.cornell.edu/drosophila/). Selected stocks were used either in the original 12 genomes project or for subsequent published genome assemblies. Originally, eight stocks were obtained from an in-house collection at the Stowers Institute (*ananassae*, *erecta*, *mojavensis*, *sechellia*, *simulans*, *virilis*, *pseudoobscura*, and *yakuba*) and eight stocks (*biarmipes*, *bipectinata*, *eugracilis*, *kikkawai*, *mauritiana*, *miranda*, *persimilis*, and *willistoni)* were obtained from the stock center when it was located at UCSD. Unfortunately, two of the eight stocks from the stock center (*kikkawai* and *miranda*) were found to be mislabeled. We were subsequently able to determine that the stock labeled *kikkawai* was in fact *triauraria*, but we were unable to determine what species the incorrectly labeled *miranda* stock was and thus removed it from our analysis. All flies were kept in standard cornmeal-agar bottles at 25°. The *D. mauritiana* reference genome was downloaded from http://www.popoolation.at/mauritiana_genome/index.html, and other reference genomes were downloaded from either FlyBase (ftp://ftp.flybase.net/genomes/) or GenBank (https://www.ncbi.nlm.nih.gov/assembly) (Table S1).

**Table 1:** Stocks sequenced in this study

Species	Stock #*^1^*
*D. ananassae*	14024-0371.13
*D. biarmipes*	14023-0361.02
*D. bipectinata*	14024-0381.07
*D. erecta*	14021-0224.01
*D. eugracilis*	14026-0451.02
*D. mauritiana*	14021-0241.01
*D. mojavensis*	15081-1352.22
*D. persimilis*	14011-0111.01
*D. pseudoobscura*	14011-0121.94
*D. sechellia*	14021-0248.01
*D. simulans*	14021-0251.006
*D. triauraria*	14028-0691.9
*D. virilis*	15010-1051.87
*D. willistoni*	14030-0811.00
*D. yakuba*	14021-0261.01

1 Stocks were obtained from the Drosophila Stock Center when it was located at the University of California San Diego. The stock center is now located at Cornell University.

### DNA isolation and quantification

Either virgin or non-virgin females were collected under CO_2_ and immediately frozen at –70° for at least 1 hr before DNA isolation. DNA was isolated using either the Qiagen Blood & Cell Culture DNA Mini Kit or by a phenol protocol (Table S1). For the Blood & Cell Culture kit (column-based isolation) 60–100 frozen females were placed in two 1.5-mL Eppendorf tubes and frozen in liquid nitrogen before being homogenized using a pestle in 250 µl of Buffer G2 with 200 µg/ml RNase A. 700 µl of Buffer G2 with RNase A, and 50 µl of 20 mg/mL proteinase K was then added to each tube, followed by incubation at 50° for 2 hr. Each tube was spun at 5k RPM for 5 min, then the supernatant was removed and placed in a new 1.5-mL Lo-Bind Eppendorf tube and vortexed for 10 sec. The supernatant from both tubes was then transferred onto the column and allowed to flow through via gravity. The column was washed 3x with wash buffer and eluted twice with 1 mL of elution buffer into 2 separate 1.5-mL Lo-Bind tubes. 700 µl of isopropanol was added and mixed via inversion before being spun at 14,000 RPM for 15 min at 4°. The supernatant was removed and the pellet was washed with freshly prepared 70% ethanol, then centrifuged at 14,000 RPM for 10 min at 4°. The supernatant was removed and 25 µl of nuclease-free water was added to each tube and allowed to sit at room temperature for 2 hr. Both tubes were then combined and stored at 4°.

For phenol-based DNA extractions, 60–100 frozen flies were transferred to a 2-mL Kontes Dounce homogenizer (VWR #KT885300-0002) on ice in 1 mL homogenization buffer (0.1 M NaCL, 30 mM Tris-HCl pH 8.0, 10 mM EDTA, 0.5% Triton X-100). Flies were dounced 4–5x with each pestle (first the looser pestle A, then the tighter pestle B). Homogenate was transferred to a 1.5-mL Eppendorf tube on ice using a wide-bore pipet tip. Homogenizer was rinsed with 500 μL homogenization buffer and combined with homogenate. Debris was pelleted by centrifugation for 1 min at 500x g at 4°.

A wide-bore pipet tip was used to transfer supernatant containing nuclei in suspension to a clean tube. Nuclei were pelleted by centrifugation for 5 min at 2000x g at 4°. Supernatant was carefully decanted and the pelleted nuclei were resuspended in 200 μL homogenization buffer by pipetting with a large-bore tip. Nuclei were transferred to a clean tube and lysed by adding 1.268 mL extraction buffer (0.1 M Tris-HCl pH 8.0, 0.1 M NaCl, 20 mM EDTA), 1.5 μL proteinase K (20 mg/mL, Life Technologies AM2548), and 30 μL 10% SDS. Lysis was aided by gently swirling and rocking, followed by incubation at 37° for 2–4 hr without agitation.

Lysed nuclei were extracted twice with an equal volume of phenol/chloroform/isoamyl alcohol pH 8.0 (Amresco K169-100ML). Extraction was mixed gently on a rotator for 5 min then centrifuged 5 min at 5000x g at room temperature. The upper aqueous phase was transferred to a clean tube and the extraction repeated as above with chloroform (Sigma C2432). The final upper aqueous phase was transferred to a clean tube and precipitated by adding 0.1 volume 3M NaOAc (mixed gently) and 2 volumes absolute ethanol (mixed by gentle inversion). A wide-bore pipette tip was used to transfer the DNA (a white, stringy clump) to a clean tube. (Note: If high-quality low-retention tips are not available, it is recommended to Sigmacote the tips to prevent the gDNA from adhering to the plastic.) Excess ethanol was removed by pipetting. DNA was washed with 500 μL 70% ethanol and centrifuged briefly at low speed with a tabletop centrifuge. Supernatant was removed by pipetting and DNA was allowed to air dry ∼10 min before resuspending in 100 μL TE pH 8.0 at 4° overnight.

### Nanopore library preparation, sequencing, and base calling

Libraries were prepared using the Ligation Sequencing Kit 1D (Oxford Nanopore) either according to or with slight modifications to the manufacturer’s protocol. To begin the prep an average of 2.3 µg of DNA was used, which is higher than the 400 ng recommended by the manufacturer. Water or TE was added to DNA for a total volume of 46 µL. For 4 of 21 library preps (Table S1), the FFPE repair and dA-Tailing steps were combined in the following reaction mix: 46.5 µL of genomic DNA in TE, 3.5 µL of UltraII EP Buffer (NEB), 3.5 µL of FFPE DNA Repair Buffer (NEB), 3 µL of UltraII EP Enzyme (NEB), 3 µL of FFPE Repair Mix (NEB), and 0.5 µL of 100x NAD^+^ (NEB). The combined reaction was prepared in a 200-µL PCR tube and run at 20° for 1 hr followed by 65° for 30 min in a thermocycler. After cleanup and adapter ligation, 75 µL of library (note that all 15 µl of adapter-ligated DNA, not the 12 µL recommended by the manufacturer, was included in the final library), including Library Loading Beads, were loaded onto an R9.4 flow cell containing at least 800 active pores and run for 48–72 hr, or until no pores were available, on a MinION sequencer (Oxford Nanopore). Flow cells were restarted 3–6 times during a run in order to increase the number of pores in strand at any given time. Separate flow cells were used for each species. Nine species each utilized a single flow cell while two flow cells were used for the following six species: *D. virilis*, because of its large genome size; *D. simulans*, because of low read output on the first run; *D. bipectinata*, *D. erecta*, *D. eugracilis*, and *D. mojavensis*, because of a substandard library kit on the first run that produced fewer and shorter reads than expected (Table S1). Base calling was completed using Albacore Sequencing Pipeline Software version 2.1.0 (Oxford Nanopore) with default settings, and fastq files for either all reads or only those that passed the default quality filter (quality score ≥7) were combined for assembly and polishing (Table 2, Table S2).

**Table 2:** Base-called reads used for genome assembly. *^1^*

	Depth of coverage			All reads		Reads >1 kb		Reads >10 kb		
Species	All reads	Number of reads >1 kb	Number of reads >10 kb	Total number of reads	Average length (bp)	Number of reads	Average length (bp)	Number of reads	Average length (bp)	Longest read (bp)
*D. ananassae*	44.8	42.9	20.1	2,085,829	4,227	1,409,289	5,990	252,071	15,690	110,391
*D. biarmipes*	28.8	27.5	12.9	1,375,651	4,102	861,668	6,232	166,579	15,155	101,492
*D. bipectinata*	22.6	20.5	9.9	1,590,181	2,909	716,348	5,861	109,688	18,473	279,705
*D. erecta*	31.7	30.6	17.5	1,022,546	4,924	658,736	7,373	151,078	18,358	176,712
*D. eugracilis*	22.5	21.2	8.2	1,424,426	3,617	905,481	5,369	117,316	15,923	152,351
*D. mauritiana*	32.6	32.1	17.1	852,775	6,045	717,219	7,066	175,669	15,402	93,106
*D. mojavensis*	45.4	44.0	23.5	1,471,959	5,129	1,063,822	6,871	189,302	20,659	245,241
*D. persimilis*	34.5	33.6	15.5	1,386,759	4,910	1,072,290	6,179	199,330	15,304	113,218
*D. pseudoobscura*	33.3	31.7	13.8	1,417,469	3,936	905,571	5,868	154,234	15,000	104,401
*D. sechellia*	23.9	23.8	16.9	390,359	10,200	366,174	10,828	110,941	25,347	254,031
*D. simulans*	30.2	30.0	25.1	389,278	12,393	311,889	15,346	140,196	28,532	309,608
*D. triauraria*	18.8	18.7	13.1	409,756	9,634	379,831	10,338	120,668	22,876	238,837
*D. virilis*	22.2	21.8	11.9	1,209,939	5,969	1,017,016	6,976	189,572	20,402	282,795
*D. willistoni*	28.5	26.2	12.9	1,868,768	3,132	923,706	5,816	158,505	16,757	100,073
*D. yakuba*	21.7	21.5	12.6	509,806	7,277	462,732	7,947	103,820	20,699	195,439
**Average**	**29.4**	**28.4**	**15.4**	**1,131,520**	**5,894**	**771,222**	**6,931**	**154,466**	**18,693**	n/a

1 All reads listed had quality scores ≥7.

### Illumina library preparation and sequencing

DNA for Illumina sequencing was prepared from 10 males using a Qiagen Blood and Tissue Kit (Table S3). Briefly, flies were frozen at –70° for at least 1 hr before DNA extraction following the manufacturer’s instructions. Genomic DNA was sonicated using the Covaris S220 and libraries were constructed on a Perkin Elmer Sciclone G3 NGS Workstation using the KAPA HTP Library Prep kit (KAPA Biosystems, Cat. No. KK8234) and NEXTflex DNA barcodes (Bioo Scientific, Cat No. NOVA-514104). Post-amplification size selection was performed on all libraries using a Pippin Prep (Sage Science). Resulting libraries were quantified using an Agilent 2100 Bioanalyzer plus an Invitrogen Qubit 2.0 Fluorometer and then pooled. Sequencing was performed on an Illumina NextSeq 500 instrument as 150 bp on a high-output, paired-end flow cell. Illumina NextSeq Real Time Analysis version 2.4.11 and bcl2fastq2 v2.18 were run to demultiplex sequencing reads and to generate FASTQ files.

### Genome assembly, polishing, and quality evaluation

Minimap2 (Version 2.1-r311) (Li 2018) and miniasm (version 0.2-r168-dirty) (Li 2016) were used to assemble either all reads or only those reads with quality scores ≥7 (Table 3). Polishing was completed using either Racon (Vaser *et al.* 2017) or Pilon (Walker *et al.* 2014) alone or in combination (Table 4). Dot plots were generated with nucmer and mummerplot (Delcher *et al.* 1999); for clarity, only contigs or scaffolds >100 kb were plotted. Assemblies using only reads that passed quality filter were subjected to either four iterations of polishing with Racon, six iterations of polishing with Pilon, or three iterations of polishing with Racon followed by three iterations of polishing with Pilon. QUAST-LG (Mikheenko *et al.* 2018) was used to compare the miniasm assemblies with the published reference genomes and run on contigs >10 kb with the fragmented and large options (Table S4). BUSCO version 2.0.1 (Simão *et al.* 2015) was used to evaluate assembly quality for all genomes using the metazoan_odb9 database, which contains 978 single-copy genes likely to be present in any metazoan genome (Table 4, Tables S5–S8). For the 10 genomes assembled in this report that were part of the original Drosophila 12 genomes, transcripts were downloaded from FlyBase and aligned to each assembly and each reference genome using BLAST (Altschul *et al.* 1997). For each species and both genome types (assembly and reference), those transcripts for which at least 90% of the transcripts aligned to the genome with at least 95% identity were counted as proper alignments. To perform SNP calling, Illumina reads were aligned using bwa version 0.7.17-r1188 (Li and Durbin 2009) to assembled genomes that had been polished with Racon and Pilon. Samtools was then used to identify SNPs and indels. Only those SNPs and indels with quality scores >220 were counted (Table 5).

**Table 3:** Assembly statistics for published assemblies using scaffold values and contig statistics of miniasm assemblies after polishing with Racon three times followed by Pilon three times

		Published assembly						miniasm assemblies using only “pass” reads				
Species	Estimated genome size (Mb)	Version	Assembly size (Mb)	Number of scaffolds	Scaffold N50 (Mb)	Number of scaffolds >100 kb	Number of scaffolds >1 Mb	Assembly size (Mb)	Number of contigs	Contig N50 (Mb)	Number of contigs >100 kb	Number of contigs >1 Mb
*D. ananassae*	196.6	1.05	231.0	13,749	4.6	100	29	188.6	371	2.6	147	44
*D. biarmipes*	195.6	2.0	169.4	5,523	3.4	95	32	184.6	666	2.8	224	32
*D. bipectinata*	204.6	2.0	167.3	5,500	0.7	124	35	160.5	570	0.6	354	26
*D. erecta*	158.9	1.05	152.7	5,124	18.7	38	8	127.7	58	16.6	29	21
*D. eugracilis*	228.9	2.0	156.9	4,946	1.0	150	38	156.4	547	1.0	256	42
*D. mauritiana*	157.9	1.0	117.7	16	21.1	12	7	131.9	272	4.7	64	28
*D. mojavensis*	166.3	1.04	193.8	6,841	24.8	51	12	162.1	122	5.0	86	39
*D. persimilis*	197.1	1.3	188.4	12,838	1.9	142	30	165.7	415	3.5	146	35
*D. pseudoobscura*	167.7	3.04	152.7	4,790	12.5	25	13	160.5	361	3.0	143	33
*D. sechellia*	166.7	1.3	166.6	14,730	2.1	101	23	133.4	109	7.4	59	23
*D. simulans*	159.6	2.02	125.0	7,619	23.5	8	6	132.2	76	7.7	61	24
*D. triauraria*	210.2	n/a	n/a	n/a	n/a	n/a	n/a	170.5	482	0.72	339	34
*D. virilis*	325.4	1.06	206.0	13,530	10.2	79	22	165.9	141	4.1	83	40
*D. willistoni*	205.4	1.05	235.5	14,838	4.5	80	38	197.7	490	1.5	270	49
*D. yakuba*	170.7	1.05	165.7	8,122	21.8	60	8	141.1	111	5.2	68	32

**Table 4:** BUSCO scores*^1^* reveal assembly quality

Species	Published assembly	miniasm	miniasm –> Racon x1	miniasm –> Racon x4	miniasm –> Pilon x1	miniasm –> Pilon x6	miniasm –> Racon x3 –> Pilon x3
*D. ananassae*	98.2	1.3	88.3	91.6	74.7	91.8	98.2
*D. biarmipes*	98.6	3.8	88.3	92	77.1	94.7	98.7
*D. bipectinata*	98.2	1.7	74.9	80.1	67.2	87.9	93.9
*D. erecta*	98.6	2.5	89.9	90.4	79.5	95.5	98.6
*D. eugracilis*	98.5	0.8	82.6	86.7	70.7	92.7	97.9
*D. mauritiana*	98.6	1.4	91	94.6	75.9	95	98.7
*D. mojavensis*	98.2	0.5	82.5	88.7	66.9	93.5	98
*D. persimilis*	96.6	0.4	80.3	85.3	62.1	90.9	98
*D. pseudoobscura*	97.0	1.7	84.1	88.8	72.3	93.9	98
*D. sechellia*	97.2	1.5	91.3	92.1	75.3	95.2	98.7
*D. simulans*	98.6	2.7	91.3	95.6	77.5	94.4	98.6
*D. triauraria*	NA	3.1	82.9	85.0	73.5	87.9	93.8
*D. virilis*	97.5	1.1	84.9	89.3	70.9	93.6	97.7
*D. willistoni*	98.4	0.6	79.7	82.8	72.2	92.1	98.1
*D. yakuba*	98.5	1.3	86.7	91.2	73.7	95.5	98.4

1 Only complete BUSCO scores for the miniasm assembly using reads that passed filter (≥7) are shown. All values are percentages. Higher scores suggest better assembly.

**Table 5:** Number of single nucleotide and indel polymorphisms after polishing

	After 4 iterations of Racon			After 6 iterations of Pilon			After 3 iterations of Racon followed by 3 iterations of Pilon		
Species	Indels	Homozygous SNPs	Heterozygous SNPs	Indels	Homozygous SNPs	Heterozygous SNPs	Indels	Homozygous SNPs	Heterozygous SNPs
*D. ananassae*	107,558	36,905	10,643	4,189	294	45,486	1,056	445	10,215
*D. biarmipes*	256,004	67,310	70,947	9,646	593	123,890	5,341	1,306	73,149
*D. bipectinata*	310,109	114,548	654,543	71,581	3,173	655,658	59,176	5,056	674,390
*D. erecta*	209,290	45,382	22,186	2,758	289	37,946	1,150	435	22,179
*D. eugracilis*	313,426	85,150	712,410	75,480	3,359	708,483	67,204	5,024	740,846
*D. mauritiana*	131,501	38,401	25,587	3,325	264	39,644	1,954	433	24,885
*D. mojavensis*	213,411	71,191	36,205	9,772	311	49,328	8,516	755	35,128
*D. persimilis*	306,714	95,957	103,767	20,144	856	130,685	15,423	1,042	76,465
*D. pseudoobscura*	266,278	66,505	20,625	5,603	298	54,530	3,265	379	19,349
*D. sechellia*	214,673	37,740	17,547	2,840	300	34,525	1,420	538	18,117
*D. simulans*	145,544	39,508	40,197	5,008	349	54,565	3,051	475	40,803
*D. triauraria*	373,410	171,450	843,725	79,228	3,461	812,711	68,228	5,745	865,396
*D. virilis*	211,137	71,644	19,972	5,503	265	48,459	4,207	757	20,272
*D. willistoni*	332,107	110,725	382,209	64,535	2,770	388,208	59,316	5,193	405,046
*D. yakuba*	283,296	92,344	28,566	4,248	424	59,967	1,839	687	27,336

### Alignment of reference contigs to Nanopore assemblies

Each reference genome was broken into contigs by separating scaffolds at every position that contained an N, and BLAST (Altschul *et al.* 1997) was used to align these to contigs from our Nanopore assembly using a custom script. We first aligned only reference contigs larger than 10 kb from reference scaffolds that contained no N’s (that had no gaps) and counted how many of these reference contigs mapped with an identity >99% to at least one assembled contig. We then aligned only reference contigs that came from reference scaffolds with two or more contigs (meaning the reference scaffold had at least one gap) one reference scaffold at a time. A gap was assumed to be filled if at least two reference contigs mapped to the same assembled contig.

### Data availability

Illumina data generated for this project are available at the National Center for Biotechnology Information (https://www.ncbi.nlm.nih.gov/) under project PRJNA427774, Nanopore reads that passed the default Albacore filter are available under project PRJNA471302. Illumina and Nanopore data for *D. triauraria* are available under project PRJNA473618. Scripts used in this project and genomes assembled in this project can be found on GitHub at https://github.com/danrdanny/Drosophila15GenomesProject/. Original data underlying this manuscript can be accessed from the Stowers Original Data Repository at http://www.stowers.org/research/publications/libpb-1269. Supplemental material available at Figshare: https://doi.org/10.25387/g3.6939758.

## Results and Discussion

### Sequencing

Nanopore sequencing runs on a flow cell containing a maximum of 2,048 pores through which single-stranded DNA passes. These 2,048 pores are contained within 512 channels, with four pores per channel. Currently, only one pore in a channel—512 total pores—are available for sequencing at a time. The sequencing control software, called MinKNOW, consigns each pore on a flow cell into one of four groups (often called mux groups) based on its performance level. Each mux group is run for a predetermined amount of time (8 hr by default), at which point sequencing switches to the next group of pores because pore quality deteriorates over time.

For this study, we generally ran a sequencing reaction for 24 hr, allowing each of the pores in groups 1–3 to run for 8 hr (pores in group 4 are not used during a run). We then stopped and restarted the flow cell, allowing pores to be reorganized into new groups, which also allows pores originally assigned to the unused group 4 to be moved back into groups 1–3. We then monitored the flow cell and restarted again as the number of active pores decreased during a run, once again reorganizing pores into new groups. Because the number of active pores deteriorates over time and the amount of data output by a flow cell drops dramatically after 24 hr, there is an advantage to keeping as many pores as possible actively sequencing, or “in strand,” at a time.

Preliminary testing in our lab revealed that using the manufacturer-recommended 400 ng of input DNA for a 1D library prep resulted in fewer than 50% of active pores in strand. This low number of active pores translated into low total data output by the flow cell. We found that starting a 1D library prep with 1–10 µg of DNA resulted in substantially more pores in strand during a sequencing run and gave higher data yields. We therefore performed all library preps in this study using 1–10 µg of starting material. Sequencing 15 Drosophila species in this manner yielded a total of 23 million sequence reads (Table S2).

After base calling, these 23 million reads, with an average read length of 4,302 bp, yielded ∼99 billion bases sequenced at an average depth of coverage of 35x (Table S2). The base calling software, Albacore, separates reads into ‘pass’ and ‘fail’ bins by default, where any read with a quality score ≥7 is identified as a read that passed filter. The 76% of reads and 85% of total bases that passed filter had an average read length of 5,894 bp and an average depth of coverage of 29x, while those that did not pass filter had an average read length of 2,680 bp (Table 2, Table S2).

To determine whether variations in certain steps of the protocol might provide increased data output, we tested three different methods of DNA extraction and preparation. First, we compared column-based and phenol extraction (see Materials and Methods). While column-based methods are more convenient and safe, phenol extractions may reduce DNA shearing and loss. Second, in an attempt to reduce DNA shearing even further through reduced pipetting, we performed phenol extractions followed by a shortened library preparation protocol in which the FFPE repair and dA-tailing steps were combined. For reads passing filter, the average read length of samples prepared by a column-based method was 4,013 bp. Average read length sharply increased to 8,931 bp in phenol extractions that followed library prep instructions, while phenol-extracted samples that combined the FFPE repair and dA-tailing steps showed an average read length of 10,389 bp. These results suggest that combining these two library preparation steps is potentially useful, but the greatest increase in read length resulted from careful phenol-based DNA isolation.

### Genome assembly and statistics

*De novo* assembly of 15 genomes can be computationally expensive since the software typically used to assemble long error-prone sequencing reads [for example, Canu (Koren *et al.* 2017) or hybrid assemblers such as DBG2OLC (Ye *et al.* 2016), which uses both short high-quality reads and long error-prone reads] requires a large number of CPU hours to complete with default settings. As an example, for assembly of the *D. melanogaster* ISO-1 genome using only reads larger than 1 kb reported in Solares *et al.* (2018), Canu took longer than 1 week on a 32-core server with 1 terabyte of memory—or at least 5,376 CPU hours. This may be cost-prohibitive for some labs. Alternatively, mapping and assembly using miniasm (a *de novo* assembler for long error-prone reads) has been shown to be highly efficient and less time-intensive (Li 2016; 2017).

A study comparing assembly quality of the *D. melanogaster* ISO-1 reference genome using different assembly approaches found that assemblies using miniasm alone gave larger contigs (N50 3.9 Mb) than Canu alone (N50 3.0 Mb) but smaller contigs than DBG2OLC alone (N50 9.9 Mb) or a merged Canu and DBG2OLC assembly (N50 18.6 Mb) (Solares *et al.* 2018). This suggests that statistics from miniasm assemblies (as assayed by N50 value alone—a measure of genome contiguity) may be comparable to some more computationally intensive long-read assembly software. We therefore chose to perform assembly using only miniasm. Each of the 15 assemblies was completed in under 1 hr using 32 CPUs on a computer with 1 Tb of RAM available (less than 32 CPU hours). While assembly statistics such as N50 are similar between miniasm assemblies and Canu assemblies, it is important to note that the quality of miniasm assemblies are much lower because Canu contains an error-correcting step (discussed below). The addition of an equivalent error-correction step to our miniasm assemblies did increase overall assembly and analysis time. Specifically, we find that one iteration of polishing with Racon takes approximately 32 CPU hours to complete, while one round of polishing with Pilon takes approximately 64 CPU hours to complete. In total, this adds approximately 288 CPU hours to a miniasm assembly, or approximately 10-fold less than the 5,000-plus CPU hours required for Canu assemblies of similar datasets.

For each species, sequenced reads were combined into two fastq files, one containing all reads and one containing only those reads that passed filter. Separate genome assemblies were then completed for each category of reads and compared. Assemblies using only reads that passed filter had larger contig N50 values in 9 of 15 cases and fewer overall contigs in 10 of 15 cases than assemblies completed using all sequencing reads (Table 3, Table S5). Because of the moderately improved N50 values and lower contig numbers, we decided to proceed with our analysis using only assemblies generated by higher quality reads. Assembly quality varied greatly among the 15 genomes, with an average contig N50 of 4.4 Mb, a maximum of 16.6 Mb (*D. erecta*), and a minimum of 0.6 Mb (*D. bipectinata*). For each species, assembly resulted in genome sizes that were smaller than the expected genome size (Bosco *et al.* 2007; Gregory and Johnston 2008; Hjelmen and Johnston 2017), with repetitive sequence likely accounting for the lower values than expected. We also compared assembly statistics for each of our *de novo* assemblies to the published assembly of each species and found that in all cases our contig N50 values were higher than those of the published assemblies but lower than the scaffold N50 values (scaffold N50 is a measure similar to contig N50, in which half of the genome is contained in linked DNA segments, or scaffolds, larger than the value given) in all but three cases (Table 3). Published assemblies contain both contigs (contiguous DNA segments) and scaffolds (one or more contigs generally separated by gaps where the DNA sequence is unclear), while the assemblies presented here contain only contigs.

We next generated dot plots to better understand how our assemblies compare to the reference genome for the 10 species sequenced as part of the original Drosophila 12 genomes project (Drosophila 12 Genomes Consortium *et al.* 2007). Although overall our assemblies correlated well with the published reference assemblies, we did observe a few inversions, rearrangements, or duplications in each plot (Figure S1). For example, a small inversion is seen between the *D. yakuba* reference chromosome *2L* and our assembly contig utg000002l, similar to other large inversions that have been reported to be segregating in this population (Llopart *et al.* 2002). Indeed, large rearrangements and inversions are observed in several of the assemblies. It is possible that these represent changes that occurred in the stock after being initially isolated for genome sequencing, they may be due to assembly errors in the initial assemblies as they were generated using relatively low-coverage data, or the stocks we sequenced, while labeled as such, may not have been the exact stock used for sequencing by the 12 genomes consortium. We also observe differences in repetitive, poorly assembled regions of the genome. For example, the X-axis of the *D. sechellia* and *D. persimilis* plots are longer than the Y-axis, likely because our assemblies have collapsed repetitive regions that the published reference genomes have not. To further evaluate these differences in dot plots, we used QUAST-LG to compare our miniasm assemblies with their published assemblies using (Mikheenko *et al.* 2018). We find that on average, our assemblies capture 83% of the sequence present in the currently published reference genomes (defined as the total number of aligned bases divided by the genome size) (Table S4).

### Assembly polishing and evaluation

While the N50 statistic has utility as a measure of genome contiguity, it is not necessarily a good indicator of genome quality. A highly contiguous genome may in fact contain many errors that make it difficult to use for downstream analysis such as gene finding or SNP calling. We did not expect our Nanopore-only assemblies to be of high quality because raw Nanopore reads have an observed error rate of 10–15% (Li 2016; Simpson *et al.* 2017; Salazar *et al.* 2017). It is, however, possible to improve assembly quality through one or more rounds of polishing, which is a process that improves assembly quality using the original sequencing reads, reads from another technology with a relatively low error rate (*e.g.*, Illumina data), or a combination of both. We therefore sought to evaluate both the quality of our initial miniasm assemblies as well as our assemblies using various combinations of polishing techniques.

Assembly quality was evaluated using BUSCO, which searches assemblies for highly conserved genes generally present in a single copy in any genome (Simão *et al.* 2015). For example, BUSCO analysis of published reference genomes for the 15 species sequenced in this study revealed that at least 96.6% of 978 highly conserved genes were present in at least one copy in each genome (Table 4, Table S5). We ran BUSCO on all 15 of our miniasm assemblies with no polishing and found an average of 1.6% (min: 0.4%, max: 3.8%) of genes were present in at least one copy, suggesting that our initial assemblies, although highly contiguous, were indeed of poor quality. This is likely due to the high error rate of the reads used to generate the assemblies, resulting in frame shifts leading to premature stop codons within open reading frames.

**Figure 2:**
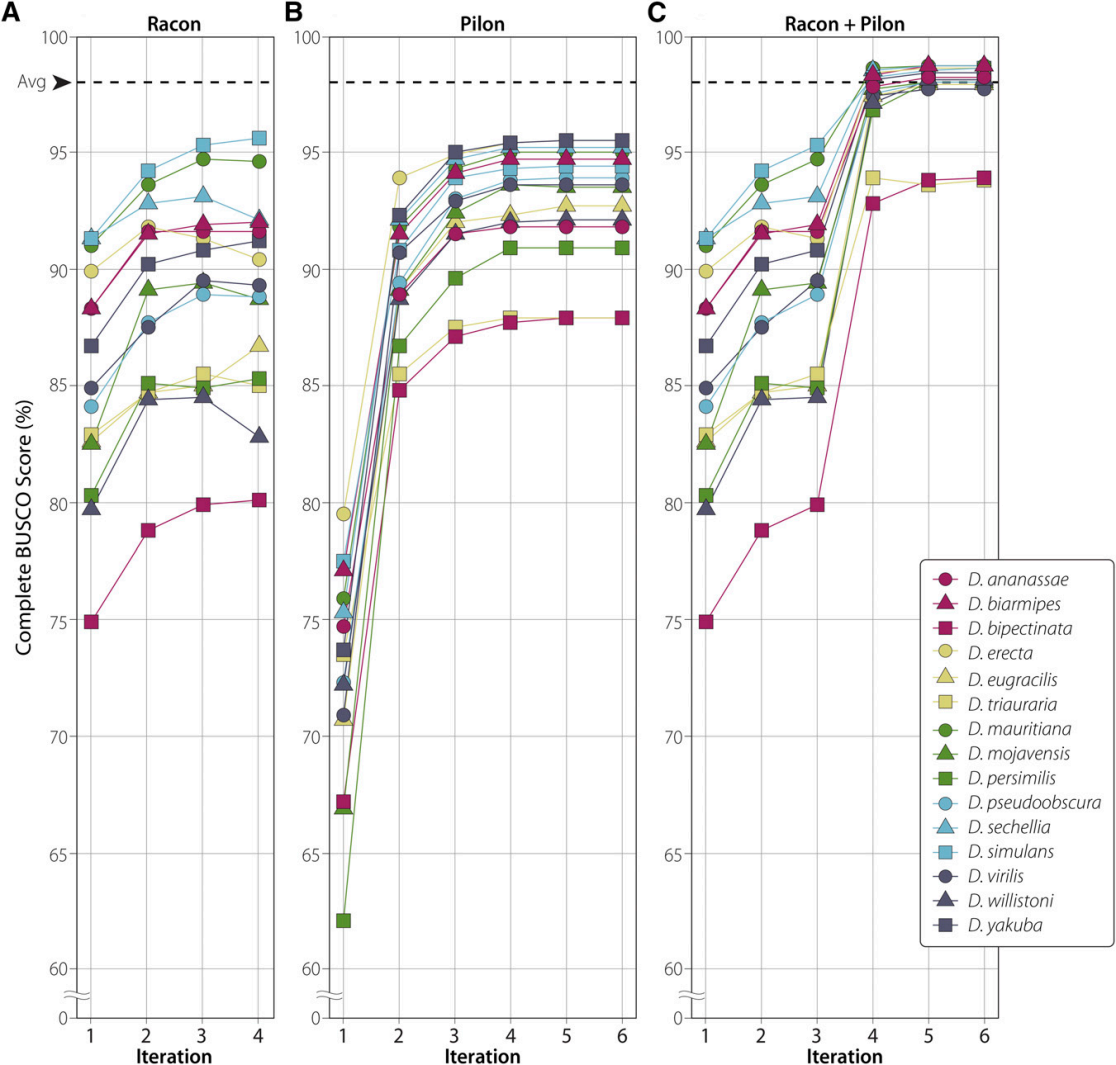
Polishing improves assembly quality. Average BUSCO score for these 15 assembled genomes was 1.6% before polishing. The dotted line in all panels represents the average BUSCO score for all 14 published reference genomes (Table 4). Full polishing results can be found in Tables S5–S8. (A) Complete BUSCO scores for four iterations of Racon alone. (B) Complete BUSCO scores for six iterations of Pilon alone. (C) Complete BUSCO scores shown for three iterations of Racon followed by three iterations of Pilon.

We then performed multiple iterations of polishing using either Racon or Pilon (Walker *et al.* 2014; Vaser *et al.* 2017). Racon, which uses only base-called Nanopore reads for polishing, improved average BUSCO scores from 1.6 to 85.2% after a single iteration (min: 74.9%, max: 91.3%). Repeat iterations of Racon alone improved scores only slightly, reaching an average of 88.9% after four iterations (min: 80.1%, max: 95.6%) (Figure 2A, Table 4, Table S6). Pilon alone, which uses only Illumina data for polishing, improved average BUSCO scores from 1.6 to 72.6% after a single iteration (min: 62.1%, max: 79.5%), and to 93.0% after six iterations (min: 87.9%, max: 95.5%) (Figure 2B, Table 4, Table S7). While BUSCO scores tended to increase with more iterations, it is important to note that BUSCO scores fell for 7 of 15 assemblies between the 3^rd^ and 4^th^ iterations of Racon, suggesting that repeat iterations of polishing may have negative impacts on assembly quality. Alignment of Illumina data to Racon-polished genomes demonstrated that polishing using Racon alone failed to completely correct poly-N indels. Furthermore, while Pilon was run for 6 consecutive iterations, there was not significant improvement in scores after 3 iterations, demonstrating a limit to polishing using a single software application.

Because both polishing techniques alone failed to achieve BUSCO scores equal to or better than the published reference genomes, we then polished using a combination of both Racon and Pilon. We first attempted to run Pilon and Racon in combination, one after the other (*e.g.*, Racon, Pilon, Racon, Pilon, etc.), but found that while BUSCO scores improved with each iteration of Pilon, they then fell with each iteration of Racon (Table S8). Because BUSCO scores plateau around 3 iterations of Racon or Pilon (see above), we wondered if combining the two approaches in tandem would result in improved assembly quality. We therefore ran three iterations of Racon followed by three iterations of Pilon and found a sustained improvement in BUSCO scores, reaching an average of 97.7% (min: 93.8%, max: 98.7%) (Figure 2C, Table 4, Table S9). Importantly, this combination of polishing resulted in BUSCO scores consistent with the currently published reference genomes for 13 of the 14 previously sequenced species. Finally, to determine if currently available gene models properly mapped to our assemblies, we mapped transcripts for the 10 assemblies that are available on FlyBase to both our assembled genomes and the currently published reference genomes. We found that on average 97.8% of all transcripts mapped to our assembled genomes, while 99.6% mapped to the published reference genome (Table S10). This suggests that while the BUSCO scores of our genomes suggest similarity between our assemblies and the reference, a small number of genes that are not considered highly conserved by BUSCO may have been improperly assembled using our approach.

To determine if high levels of heterozygosity of the stocks used for sequencing explained the lower BUSCO scores observed in the *D. bipectinata* and *D. triauraria* stocks (average of 93.9%), we aligned Illumina data from each stock to the assembly generated from three iterations of Racon and Pilon and called SNPs and indels using SAMtools (Li *et al.* 2009). On average, we observed 63,702 indels, and 769,893 heterozygous SNPs in these two stocks, compared to an average of 13,365 indels and 116,445 heterozygous SNPs in the remaining 13 stocks. This high number of SNP and indel polymorphisms reveals a high level of heterozygosity in the stocks with low BUSCO scores. However, we also observed a high number of SNP and indels in both the *D. eugracilis* and *D. willistoni* stocks, two assemblies with BUSCO scores near 98%, suggesting that heterozygosity for SNPs and indels alone does not fully explain the lower quality scores observed in *D. bipectinata* and *D. triauraria*.

These observations led us to ask why a large number of heterozygous SNPs were observed in four of our lines. Because no effort was made to isogenize these stocks before sequencing, it is possible that the high number of SNPs and indels in these lines is simply a consequence of selection for heterozygosity in these lines. However, this hypothesis would predict high levels of heterozygosity in the other sequenced stocks, which is not observed. Another possibility is that that these four lines were crossed intentionally with another stock in order to increase the fitness of the stock, or they were contaminated. Either scenario would have introduced the large amount of heterozygosity we observe.

Finally, we wondered if polishing would merge or separate contigs and thus affect assembly statistics. We found that polishing with each program independently did, on average, increase the total assembly size and contig N50 of our 15 assemblies (Table S11). Furthermore, the total number of contigs decreased for 6 of 15 species and increased for none after polishing with Racon, while total contig number remained unchanged after polishing with Pilon. Overall, this suggests that polishing using Racon or Pilon had minimal impact on assembly statistics, but clearly improved assembly quality as determined by BUSCO scores.

### Long-read data can close gaps in reference genomes

Other long-read sequencing technologies, such as PacBio, have previously been used to improve existing reference genomes (Jiao *et al.* 2017), and visual inspection of dot plots used to compare our assemblies with the reference genome suggest that our contigs could be used to link large reference scaffolds together (Figure S1). To test if our highly contiguous genomes could be used to fill gaps in the reference assemblies, we broke each reference genome into individual contigs and aligned those contigs to our assembled genomes. We performed two types of alignment. First, we identified reference contigs larger than 10 kb that contained no N’s, or unknown sequence (these contigs are generally labeled as scaffolds in many reference genomes, which may cause confusion) and identified how many of those could be placed on our assemblies with >99% identity. In doing so, we found that each reference genome contained an average of 205 singleton contigs larger than 10 kb, and that on average 95% of these could be placed on a larger contig in our assemblies (Table 6). (*D. mauritiana* was excluded from this analysis because its genome assembly is based on release 5 of the *D. melanogaster* genome and thus contains only one contig larger than 10 kb with no gaps.)

**Table 6:** Number of singleton reference contigs that could be placed on the contigs assembled in this study and the number of gaps closed between reference contigs on scaffolds with one or more gaps

Species	Number of reference scaffolds with at least one gap	Number of reference contigs aligned to assembly	Number of gaps in the reference assembly	Number of reference assembly gaps potentially closed	Percentage of reference assembly gaps potentially closed
*D. ananassae*	2,305	9,088	6,783	3,264	48%
*D. biarmipes*	1,258	3,816	2,558	1,506	59%
*D. bipectinata*	1,639	4,996	3,357	1,624	48%
*D. erecta*	1,064	3,550	2,486	1,190	48%
*D. eugracilis*	1,153	4,628	3,475	1,768	51%
*D. mauritiana*	15	12,459	12,444	10,726	86%
*D. mojavensis*	1,401	6,434	5,033	3,300	66%
*D. persimilis*	1,636	15,611	13,975	11,464	82%
*D. pseudoobscura*	956	5,551	4,595	2,298	50%
*D. sechellia*	1,558	8,253	6,695	5,431	81%
*D. simulans*	994	4,599	3,605	2,572	71%
*D. virilis*	1,101	5,953	4,852	3,242	67%
*D. willistoni*	1,728	7,248	5,520	1,862	34%
*D. yakuba*	1,162	6,562	5,400	3,704	69%
Average	1,284	7,053	5,770	3,854	61%

We then identified scaffolds from each reference genome that contained two or more contigs separated by N’s and found that the 14 reference genomes contain an average of 1,284 scaffolds with two or more contigs (min: 15, max: 2,305). For each reference scaffold, individual reference contigs were mapped to the assembled genome and the gap between mapped reference contigs was determined. A single gap was considered closed when reference contigs from the same reference scaffold were mapped to a single assembled contig. This mapping revealed that, on average, our assembled contigs closed 3,854 of 5,770, or 61%, of gaps in each of the reference genomes (Table 6).

**Figure 3:**
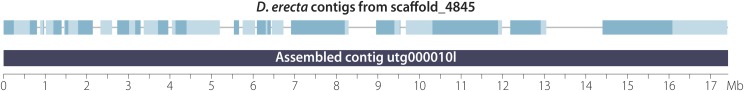
Gaps in the reference genome assembly can be closed using long-read data. This example shows that a 17.4-Mb contig (utg000010l) from our assembly (bottom) closes the gaps (top, gray lines) among 38 contigs (top, shaded boxes) from the *D. erecta* reference scaffold (scaffold_4845), potentially resolving 3.7 Mb of sequence.

A specific example of how long, contiguous assemblies may assist with reference genome correction is scaffold_4845 in the *D. erecta* reference assembly. This 22.6-Mb reference scaffold contains 75 contigs and 112,933 N’s. We broke this scaffold into its 75 contigs (numbered 0–74) and were able to place 39 of these reference contigs (numbered 23–61) onto contig utg000010l, a single 17.4-Mb contig from our assembly. These 39 contigs were placed in the same order as they appear in the reference assembly (Figure 3), but with important exceptions. Specifically, three reference contigs were placed within larger reference contigs. For example, contig 53 is a 79,102-bp contig that was placed in the middle of contig 52, a 339,371-bp contig. Similarly, contig 39 was placed within 40, and contig 60 was placed within 61. All six contigs have >99% identity to the positions that they were aligned to, suggesting either that the reference assembly incorrectly identified these contigs as unique segments of the genome or that our assembly collapsed nearby regions of the genome with high identity into single segments.

### Sequencing costs and conclusions

A major goal of this project was to determine if it was possible to create low-cost, highly contiguous genome assemblies of non-*melanogaster* Drosophila species using Nanopore sequencing. We are able to estimate an expected cost for each of our Drosophila genomes using publicly available materials and reagent prices from early 2018. Here, we used approximately one flow cell per species; when purchased individually, a single flow cell is $900 (USD) and when purchased as a pack of 48, a single flow cell is $500 (USD). A 1D library kit is $599 (USD) and will make six libraries, at a cost of $100 per genome. Reagents include FFPE enzyme, dA-tailing enzyme, ligase, and magnetic beads for cleanup. To simplify, we assume $50 in reagent and other costs per genome. Therefore, for Nanopore sequencing alone, the costs for materials and reagents range from $650–$1,050 depending on the flow cell cost. We also generated Illumina 150-bp paired-end data for polishing at an average of 64x depth of coverage (Table S3). While short read sequencing costs vary widely, we assume a cost of $250 per Drosophila genome is a reasonable estimate. Therefore, we estimate that overall sequencing and assembly costs for a highly contiguous Drosophila reference genome to range from $900–$1,300 (USD).

In summary, we have generated highly contiguous genome assemblies for 15 species of Drosophila at a materials and reagents cost of approximately $1000 (USD). Relatively low-coverage Nanopore data resulted in genome assemblies with an average contig N50 value of 4.4 Mb, and polishing with Racon and Pilon resulted in average BUSCO scores of 97.7%, a score comparable to currently published reference genomes (Figure 2, Table 3, Table 4). That we were able to generate 15 different genome assemblies at relatively low cost and in a short period of time suggests a new approach researchers may take when studying other species within the *Drosophila* genus and suggests that genome assembly using a long-read technology should now be considered the standard for studies of new Drosophila species.

It is now also feasible to consider what effort, if any, should be put forth in attempts to assemble and analyze a substantially larger number of genomes from across the entire *Drosophila* genus. The *Drosophila* genus represents at least 50 million years of evolution (Tamura *et al.* 2004; Obbard *et al.* 2012; O’Grady and DeSalle 2018) and spans a broad range of ecosystems, with the unique advantage of having a highly developed genetic toolkit available for many of its members (Ashburner *et al.* 2005; Gratz *et al.* 2013; Perkins *et al.* 2015; Stern *et al.* 2017). Low-cost, highly contiguous genome assemblies of hundreds of well-described species would foster studies of genome conservation and evolution orders of magnitude more detailed than any before and on a scale not possible in other species groups. Such work would provide a solid foundation to ensure the next 100 years of Drosophila research are as fruitful as the first 100 years have been.
